# Estrogen Receptor Alpha as a Key Target of Red Wine Polyphenols Action on the Endothelium

**DOI:** 10.1371/journal.pone.0008554

**Published:** 2010-01-01

**Authors:** Matthieu Chalopin, Angela Tesse, Maria Carmen Martínez, Didier Rognan, Jean-François Arnal, Ramaroson Andriantsitohaina

**Affiliations:** 1 INSERM, U771, CNRS UMR, 6214, Université d'Angers, Angers, France; 2 Bioinformatics of the Drug, UMR 7175 CNRS-ULP (Université Louis Pasteur-Strasbourg I), Illkirch, France; 3 INSERM U858, Université Toulouse III Paul Sabatier, CHU (Centre Hospitalier Universitaire), Toulouse, France; University of Camerino, Italy

## Abstract

**Background:**

A greater reduction in cardiovascular risk and vascular protection associated with diet rich in polyphenols are generally accepted; however, the molecular targets for polyphenols effects remain unknown. Meanwhile evidences in the literature have enlightened, not only structural similarities between estrogens and polyphenols known as phytoestrogens, but also in their vascular effects. We hypothesized that alpha isoform of estrogen receptor (ERα) could be involved in the transduction of the vascular benefits of polyphenols.

**Methodology/Principal Findings:**

Here, we used ERα deficient mice to show that endothelium-dependent vasorelaxation induced either by red wine polyphenol extract, Provinols™, or delphinidin, an anthocyanin that possesses similar pharmacological profile, is mediated by ERα. Indeed, Provinols™, delphinidin and ERα agonists, 17-beta-estradiol and PPT, are able to induce endothelial vasodilatation in aorta from ERα Wild-Type but not from Knock-Out mice, by activation of nitric oxide (NO) pathway in endothelial cells. Besides, silencing the effects of ERα completely prevented the effects of Provinols™ and delphinidin to activate NO pathway (Src, ERK 1/2, eNOS, caveolin-1) leading to NO production. Furthermore, direct interaction between delphinidin and ERα activator site is demonstrated using both binding assay and docking. Most interestingly, the ability of short term oral administration of Provinols™ to decrease response to serotonin and to enhance sensitivity of the endothelium-dependent relaxation to acetylcholine, associated with concomitant increased NO production and decreased superoxide anions, was completely blunted in ERα deficient mice.

**Conclusions/Significance:**

This study provides evidence that red wine polyphenols, especially delphinidin, exert their endothelial benefits *via* ERα activation. It is a major breakthrough bringing new insights of the potential therapeutic of polyphenols against cardiovascular pathologies.

## Introduction

Epidemiological studies have enlightened that women have lower cardiovascular risk than men, and this protection progressively disappears after menopause. These studies (protection in premenopausal women) suggest and experimental studies (prevention of atheroma development in animals) demonstrate a major atheroprotective action of 17-β-estradiol (E_2_) [1], [2]. E_2_ actions are essentially mediated by two molecular targets: estrogen receptor alpha (ERα) and beta (ERβ), but the former appears to mediate most of the actions of E_2_ on the cardiovascular system [1], [2]. Endothelium represents a well recognized target of E_2_, which elicits several beneficial actions as increased NO production [1]–[3] subsequent to activation of endothelial NO synthase (eNOS) *via* a G-protein [4], and ERK and phosphatidylinositol-3-kinase pathways.

Epidemiological studies reported a greater reduction in cardiovascular risk and greater vascular protection associated with diet rich in polyphenols, including those from red wine [5]. We have previously shown that Provinols™, a polyphenolic extract from red wine, and delphinidin, an anthocyanin pharmacologically active and found in the total extract, induce an increase of endothelial NO production leading to endothelium-dependent relaxation [6], [7], even in pathophysiological contexts as hypertension, metabolic syndrome or stroke [8]–[10], and restore endothelial function [11]. Although intracellular pathways involved in the endothelial effects of polyphenols are partially described (increase of intracellular calcium, activation of tyrosine kinases, for instance) [7], the molecular targets of these polyphenols remain unknown. It has to keep in mind that numerous molecules contained in red wine polyphenols including resveratrol might act on synergistic ways, in addition to their antioxidant properties, by acting as agonists of sirtuin in order to increase life span and to silence metabolic and physiological disturbances often associated with endothelial NO dysfunction [12], [13].

Evidences in the literature have enlightened, not only structural similarities between estrogens and polyphenols known as phytoestrogens, but also in their vascular effects with regard to endothelial NO production. Indeed, it has been reported that the phytoestrogen genistein produces acute NO-dependent dilation of human forearm vasculature with similar potency to E_2_ [14]. Also, genistein induces a late but sustained activation of the eNOS system *in vitro* [15]. Moreover, chronic administration of genistein improves endothelial dysfunction in spontaneously hypertensive rats that involves eNOS, caveolin and calmodulin expression and NADPH oxidase activity [16].

Red wine polyphenols do not contain genistein and no direct evidence for the nature of the receptor triggering the effect of red wine polyphenols receptors in endothelial cells has been demonstrated. Nevertheless, the aim of this work was to investigate the hypothesis that ERα is one of the targets involved in the vasculoprotective effects of Provinols™ and delphinidin. For this, we first studied the endothelium-dependent relaxation to polyphenols in aortas from both ERα Knock-Out (KO) and Wild Type (WT) mice, and then we analyzed molecular pathways associated with NO production in endothelial cells stimulated by Provinols™ and delphinidin by silencing ERα activity or expression either with pharmacological inhibitor or siRNA, respectively. We also studied binding assay and molecular modelling of interaction between delphinidin and ERα. Finally, we tested the physiological relevance of our findings *in vivo* by testing the effect of short term oral treatment of ERα KO and WT mice with Provinols™ with respect to endothelial NO response.

## Results

**Figure 1:**
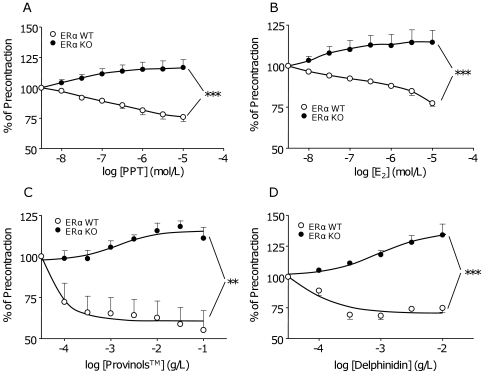
ERα mediates endothelium-dependent relaxation induced by agonists, Provinols™ and delphinidin. Concentration-effect curves to increasing concentrations of PPT (A), 17-β estradiol (E_2_) (B), Provinols™ (C) or delphinidin (D) in aortic rings, with functional endothelium precontracted with U46619, taken from ERα Wild Type (open circles) and Knock-Out (filled circles) mice (n = 5–6). **P<0.01, ***P<0.001.

The role of ERα in the endothelium-dependent relaxation to Provinols™ and to delphinidin was evaluated by using vessels taken from ERα WT and KO mice. First, we tested the ability of ERα agonists such as E_2_, which acts on both ERα and ERβ isoforms, and 1,3,5-tris(4-hydroxyphenyl)-4-propyl-1H-pyrazole (PPT), which is specific for ERα, to activate the endothelium. This was demonstrated by the capacity of the two ERα agonists to induce relaxation in aortas from ERα WT but not from KO mice in the presence of functional endothelium only (Figure 1A and 1B). The concentration of E_2_ to elicit maximal relaxation was in accordance with that reported by Li *et al* [17] in the same vessels. As previously described by our group, red wine polyphenols and delphinidin are able to induce endothelium-dependent relaxation in mice aortas. Interestingly, the vasorelaxant effect of these two polyphenols was found in aortas from ERα WT mice (Figure 1C and 1D), but was completely abolished when ERα is deleted. In ERα deficient mice, a slight contraction to Provinols™ and delphinidin were even detected (Figure 1C and 1D).

**Figure 2:**
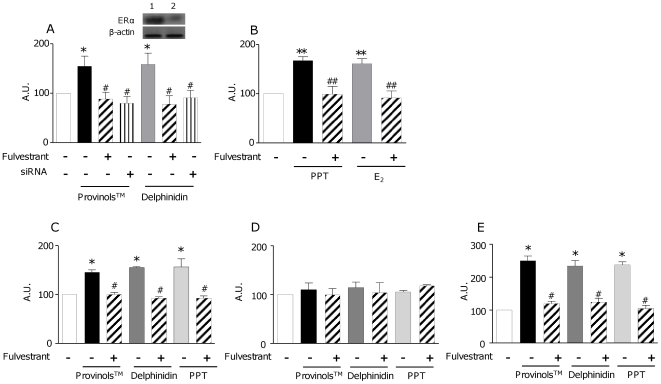
ERα activation induces endothelial NO release. NO production induced by Provinols™, delphinidin, PPT and 17-β estradiol (E_2_) in endothelial cells and consequences of ERα pathway inhibition with either Fulvestrant or siRNA directed against ERα (siRNA). EaHy 926 cells (A and B) or aortic endothelial cells from either ERα Wild-Type (WT) mice (C), ERα Knock-Out (KO) mice (D) or from Swiss mice (E) were treated for 10 minutes with Provinols™ (10^−2^ g/L), delphinidin (10^−2^ g/L), PPT (10^−5^ M) or E_2_ (10^−5^ M) and this in the presence or in the absence of either Fulvestrant (30 nM) (hatched bars) or after knocking down the receptor with siRNA (siRNA) (striped bars). Then NO production was assessed by electronic paramagnetic resonance. In panel A, insert shows Western blot for ERα in the control (lane 1) or ERα siRNA (lane 2)-treated Eahy endothelial cells. *P<0.05, **P<0.01, ***P<0.001 *vs* non treated; #*P*<0.05, ##*P*<0.01 *vs* Provinols™, delphinidin (A, C and D), PPT or E_2_ alone (B and D), (n = 4–6).

These data suggest the involvement of ERα in the endothelium-dependent relaxation in response to the two polyphenols used. Then, we assessed if the endothelium-dependent relaxation evoked by ERα stimulation is due to an increase in NO production. For this we stimulated the human endothelial cell line, EaHy 926, either with Provinols™ or delphinidin for 10 minutes in presence or in absence of Fulvestrant, an ERα pharmacological antagonist, or with a siRNA directed against ERα. Provinols™ and delphinidin were used at maximally active concentration to induce relaxation in the rat aortic rings and to increase cytosolic calcium in endothelial cells, as previously described [7], [18]. Both Provinols™ and delphinidin were able to induce an increase in NO production. However, when ERα was silenced either by Fulvestrant or siRNA, the increase of NO production induced by Provinols™ and delphinidin was completely prevented (Figure 2A). As a positive control for ERα activation-induced NO production, we used PPT and E_2_. Both agonists were able to enhance NO production, and this effect was abolished when ERα was antagonized by Fulvestrant (Figure 2B). As EaHy 926 are derived from human umbilical vein endothelial cells, we extracted and cultured endothelial cells from aortas taken either from ovariectomized ERα WT or KO mice, or from ovariectomized Swiss mice, using the method described by Kobayashi *et al.* [19]. Cells were then stimulated with Provinols™, delphinidin and PPT, in absence or in presence of Fulvestrant, using the same protocol as for EaHy 926. Interestingly, in aortic endothelial cells taken either from ERα WT mice or Swiss mice, Provinols™, delphinidin and PPT were all able to induce NO production (Figure 2C and 2E). The ability of the three compounds to stimulate NO release was completely blunted in the aortic endothelial cells extracted from ERα KO mice (Figure 2D) or in aortic endothelial cells taken from ERα WT mice and Swiss mice in the presence of Fulvestrant (Figure 2C and 2E).

**Figure 3:**
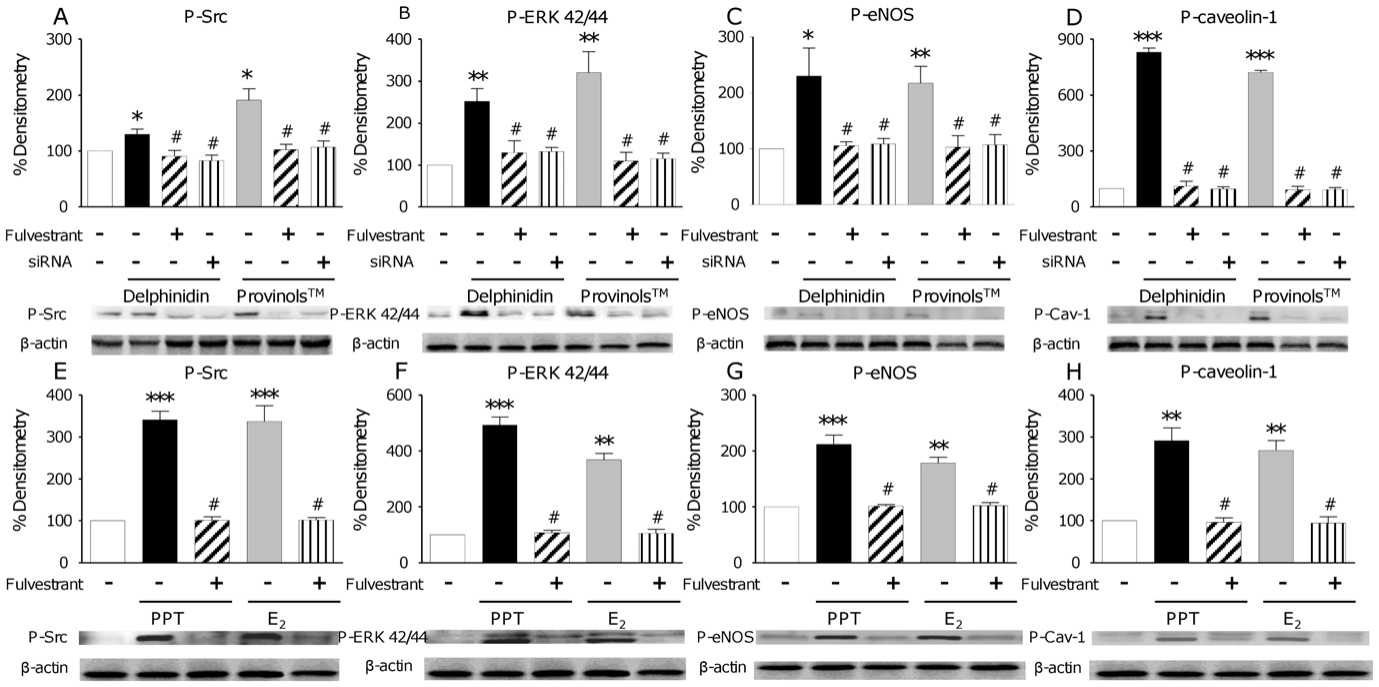
ERα activation induces phosphorylation of NO signaling proteins. Activation of NO pathway induced by Provinols™, delphinidin, PPT and 17-β estradiol (E_2_) in endothelial cells and consequences of ERα pathway inhibition with either Fulvestrant or siRNA directed against ERα (siRNA). EaHy 926 cells were treated for 10 minutes with Provinols™ (10^−2^ g/L), delphinidin (10^−2^ g/L), PPT (10^−5^ M) or E_2_ (10^−5^ M) and this in the presence or in the absence of either Fulvestrant (30 nM) (hatched bars) or after knocking down the receptor with siRNA (siRNA) (striped bars). Then cells were lysed to perform Western Blot analysis. *P<0.05, **P<0.01, ***P<0.001 *vs* non treated; #*P*<0.05, ##*P*<0.01 *vs* Provinols™, delphinidin (A, B, C and D), PPT or E_2_ alone (E, F, G and H), (n = 4–6).

Recently, an increase of NO production *via* the interaction of a molecular pathway involving the phosphorylation of Src, ERK1/2 and eNOS on the Ser1177 has been reported with regard to resveratrol [20]. NO pathway was then investigated in order to decipher the molecular mechanisms underlying ERα-associated NO increase and the subsequent vasodilatation induced either by Provinols™, delphinidin or by PPT and E_2_. We also analysed caveolin-1 expression, a protein that segregates the inactive form of eNOS on the cellular membrane and modulates eNOS activity. We demonstrated that both Provinols™ and delphinidin increased the phosphorylation of Src, ERK1/2 and eNOS Ser 1177, as well as of caveolin-1, in human endothelial cells. Moreover, when ERα was blocked or silenced, the activation of this pathway was completely blunted (Figure 3A to 3D). In addition, the same effects were observed after PPT or E_2_ treatment in the sense that the two ER agonists increased phosphorylation of Src, ERK1/2, eNOS and caveolin-1. Furthermore, when ERα was antagonized with Fulvestrant, neither PPT nor E_2_ induced phosphorylation of these enzymes (Figure 3E to 3H), suggesting the involvement of an ERα-dependent mechanism. Even if it has already been shown that both Provinols™ and delphinidin are able to induce NO production in endothelial cells [18], and that estrogens are also able to increase NO production *via* ERα [21], here we demonstrate for the first time the direct link between the ability of Provinols™ and delphinidin to stimulate NO pathways leading to endothelial NO production through ERα activation.

**Figure 4:**
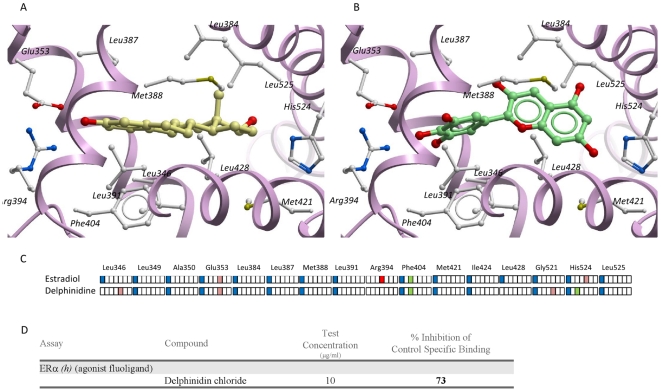
Binding assay and docking show direct interaction between delphinidin and ERα activator site. Binding mode of 17-β estradiol (E_2_) (panel A, X-ray structure) and delphinidin (panel B, docking model) to the ligand-binding domain of the ERα (1a52 PDB entry). The receptor backbone is displayed by solid ribbons. Ligand-contacting side chains are displayed by white ball and sticks. Carbon atoms of receptor-bound E_2_ and delphinidin are in yellow and green, respectively. In panel C, the ligand-receptor interactions for both compounds are encoded by an interaction fingerprint converting into a 7 bit string the interaction of the ligand with each residue of the binding site using the following color-coding: blue, apolar contact; green: aromatic interaction; red: hydrogen-bond. Eventually, D represents results of a binding assay of delphinidin on ERα. E_2_ was used as control agonist for ERα and delphinidin was used at concentration exerting endothelial effects shown before (10^−2^ g/L).

To verify the direct interaction of red wine polyphenols with ERα and to exclude the implication of other factors, binding assay between delphinidin and ERα was performed. Binding assay showed that delphinidin exerts 73% of specific inhibition against E_2_ on ERα (Figure 4D). Furthermore, we performed a docking study of delphinidin on ERα. The predicted binding mode of the ligand-binding domain on ERα is relatively similar to that observed in the X-ray structure of the ERα with E_2_ (Figure 4A and 4B). The three aromatic rings undergo significant apolar contacts with hydrophobic residues located at the centre of the binding site (Leu349, Ala350, Leu384, Leu387, Met388, Leu391, Phe404, Met421, Ile424, Leu428, Leu525) (Figure 4C). An aromatic edge-to-face interaction is also engaged with the phenyl ring of Phe404, as for E_2_. Last, a strong H-bond anchors delphinidin and E_2_ to a polar residue (Glu353) at one end of the binding site. A significant difference with the E_2_ binding mode is the loss of two H-bonds to Arg394 and His524, which are partly compensated by novel interactions to Leu346 (H-bond to the backbone oxygen atom) and His524 (aromatic interaction). These results indicate that delphinidin, like E_2_, interact directly with ERα.

**Figure 5:**
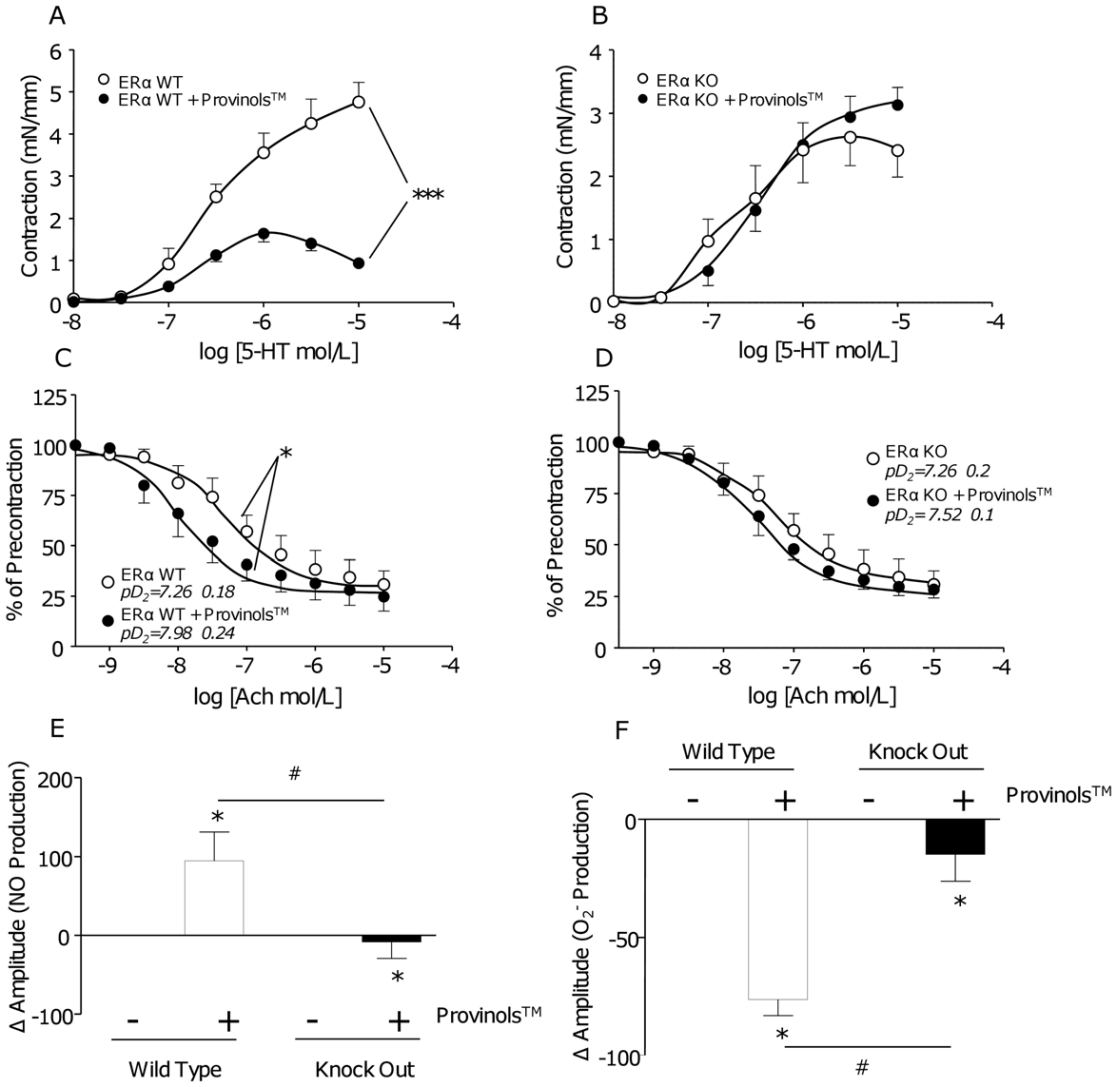
Involvement of ERα in vascular effects induced by an oral treatment with Provinols™. Concentration-effect curves to increasing concentrations of serotonin (5-HT) in aortas, with endothelium, from ERα Wild Type (WT) (A) or Knock-Out (KO) (B) mice treated either with control diet (open circles) or diet containing Provinols™ for 2 weeks at 20 mg/kg/day (filled circles, ***P<0.001, n = 6). Concentration-effect curves to increasing concentrations of acetylcholine (Ach) in aortas with endothelium, pre-contracted at 80% of the maximal contraction with U46619 from ERα WT (C) or KO (D) mice treated either with control diet (open circles) or diet containing Provinols™ for 2 weeks at 20 mg/kg/day (filled circles) (*P<0.05, n = 6). Quantification of NO (E) and O_2_^−^ production (F) in mesenteric arteries from ERα WT or KO mice receiving either standard diet or diet containing Provinols™ (*P<0.05 *vs* control diet; #*P*<0.05 KO *vs* WT; n = 5).

Finally, we show that short term oral administration of Provinols™ decreased contraction to serotonin (5-HT) in the presence of functional endothelium (Figure 5A) and enhanced the sensitivity to acetylcholine endothelium-dependent relaxation in aorta from ERα WT mice (Figure 5C) in association with increased NO production (Figure 5E) and reduced superoxide anions in mesenteric arteries (Figure 5F). All of these effects of oral administration of Provinols™ were completely blunted in ERα deficient mice (Figure 5B, 5D–5F). These results suggest that ERα triggers the *in vivo* effects of Provinols™ and demonstrate for the first time the physiological relevance of this receptor.

## Discussion

The present study identifies ERα as the, or at least one of, key receptor transducing vascular effects exerted by red wine polyphenols, particularly delphinidin with respect to NO production. Indeed, E_2_ and PPT, as well as Provinols™ and delphinidin, are able to activate molecular pathways, involving Src, ERK1/2, eNOS and caveolin-1 phosphorylations, by a mechanism that required ERα activation, with subsequent increase of endothelial NO production and endothelium-dependent vascular relaxation. Moreover, using a binding assay and a docking, we showed that delphinidin fits on ERα's activation site. Most importantly, evidence is provided that ERα triggers the *in vivo* effects of Provinols™ with respect to improvement in endothelial function given by the concomitant increase in NO and decrease in O_2_^−^ superoxide anions releases in vessels. The later demonstrate for the first time the physiological relevance of this receptor in triggering the vascular protection induced by red wine polyphenols.

Red wine contains a wide variety of polyphenols, which derive mainly from grape solids (skin and seeds) and can be divided in two classes, flavonoids and non-flavonoids. Although, the non-flavonoid, resveratrol has been reported to trigger some of the beneficial effects of red wine polyphenols including the activation of sirtuin and NO pathway, we have focused our attention on the flavonoid components of the red wine polyphenols such as delphinidin. Indeed, our previous study looking at the possible active principles that support the endothelial NO-dependent relaxation produced by red wine polyphenols, including Provinols™, demonstrate that anthocyanins and oligomeric-condensed tannins exhibit a pharmacological profile comparable to the original extract, the most potent being delphinidin [6]. Beside, delphinidin has been reported to induce endothelial NO release *via* an increase of cytosolic Ca^2+^ in endothelial cells [7] and protects against endothelial cell apoptosis acting through NO pathway [22].

The major aim of this work was to investigate the hypothesis that ERα is one of the targets involved in the vasculoprotective effects of Provinols™ and delphinidin. Firstly, we report the existence of an endothelium-dependent relaxation associated with ERα-stimulation, c-Src/ERK1/2-mediated activation of eNOS, with consequent endothelial NO release *via* a non-genomic mechanism at the same range of concentration than that reported by Li *et al.* [17]. Secondly, the most important novel observation is that the endothelium-dependent relaxation to Provinols™ and delphinidin found in aortas from ERα WT is completely abolished when ERα is deleted. Interestingly, both compounds elicit contraction in endothelium-denuded arteries taken from ERα deficient mice suggesting that the molecular targets of the two compounds on the smooth muscle are different to ERα (data not shown). Moreover, the ability of the two compounds to enhance the rapid release of NO (10 min) from cultured endothelial cells associated with phosphorylation of Src, ERK1/2, eNOS and caveolin-1 is blunted after silencing ERα either by Fulvestrant or siRNA. Finally, the capacity of Provinols™ and delphinidin to increase NO in mouse aortic endothelial cells was not only abolished in the presence of Fulvestrant and most likely when these cells were taken from ERα deficient mice. Altogether, these data demonstrate the direct link between the ability of Provinols™ and delphinidin to stimulate NO pathways leading to endothelial NO production through ERα activation. Very recently, it has been reported that the stilbene, resveratrol, rapidly activates MAPK signalling through ER localized in a “signalosome complex” at the plasma membrane and that may couple to G proteins, activate MEK1 and cause the release of Ca^2+^ accounting for NO release in endothelial cells [23]. However, the exact nature of the receptor isoform involved, ERα or ERβ, has not been directly assessed in their study although it is now well accepted that ERα is necessary in the response of E_2_ on endothelial NO production [2].

In the present study, direct interaction of delphindin with ERα excluding the implication of other factors is demonstrated by the capacity of this compound to exert 73% of specific inhibition against E_2_ on ERα. Furthermore, we perform a docking study of delphinidin on ERα. The predicted binding mode of the ligand-binding domain on ERα is relatively similar to that observed in the X-ray structure of the ERα with E_2_.

Altogether, these data provide evidence that red wine polyphenols and delphinidin in particular, through direct interaction with ERα, activate molecular pathways including Src, ERK1/2, eNOS, leading to endothelial NO production, accounting for vasorelaxation.

Finally, we demonstrate that the ability of oral administration of Provinols™ to decrease contraction to serotonin in the presence of functional endothelium and to improve endothelium-dependent relaxation in aorta from ERα WT mice in association with increased NO production and reduced superoxide anions in mesenteric arteries are completely blunted in ERα deficient mice. These data strongly suggest that ERα triggers the *in vivo* effects of Provinols™ and demonstrate for the first time the physiological relevance of this receptor. One can advance the hypothesis that ERα might by the or one of the molecular target(s) triggering the beneficial effects of dietary supplementation of Provinols™ on obesity-associated alterations with respect to metabolic disturbances and cardiovascular functions recently reported in Zucker fatty (ZF) rats [24]. Further studies should be conducted in order to evaluate the role of other pathways that may be involved in cardiovascular effects induced by red wine polyphenols including ERβ or cyclooxygenase pathways.

The findings that vascular protection induced by red wine polyphenols, and in particular by delphinidin, requires ERα activation is a major breakthrough in understanding the therapeutic potential of polyphenols in cardiovascular pathologies. These properties of red wine might explain the prevention of ischemic heart disease [24], [25], stroke [10] and metabolic diseases [24], in different experimental models.

## Methods

Provinols™ was obtained from Société Française des Distilleries (Vallon Pont d'Arc, France) and delphinidin was purchased from Extrasynthèse (Genay, France). The university of Angers ethical committee approved the present protocol. All animal studies were carried out using approved institutional protocols and were conformed the Guide for the Care and Use of Laboratory Animals published by US National Institutes of Health (NIH Publication No. 85–23, revised 1996). Methods for vascular reactivity performed in mice [26], [27] and endothelial cells extraction and culture were set up as previously described [19]. Methods for RNA interference and transient transfection to silence ERα were adapted from Agouni *et al.* [26]. NO and O_2_^−^ spin trapping and electronic paramagnetic resonance (EPR) studies and Western blotting were conducted as previously described [26]. Binding assay was performed by CEREP (Paris, France) using fluorescence polarization methods in human recombinant Sf9. Delphinidin was docked on ERα using default settings of the GOLD4.0 program [28]. Additional details of the methods used are provided in the Supplemental Data file (Methods S1).

## Supporting Information

Methods S1 (0.04 MB DOC)

## References

[1] Mendelsohn ME, Karas RH (2005). Molecular and cellular basis of cardiovascular gender differences.. Science.

[2] Arnal JF, Scarabin PY, Tremollieres F, Laurell H, Gourdy P (2007). Estrogens in vascular biology and disease: where do we stand today?. Curr Opin Lipidol.

[3] Kim KH, Moriarty K, Bender JR (2008). Vascular cell signaling by membrane estrogen receptors.. Steroids.

[4] Wyckoff MH, Chambliss KL, Mineo C, Yuhanna IS, Mendelsohn ME (2001). Plasma membrane estrogen receptors are coupled to endothelial nitric-oxide synthase through Galpha(i).. J Biol Chem.

[5] Renaud SC, Gueguen R, Conard P, Lanzmann-Petithory D, Orgogozo JM (2004). Moderate wine drinkers have lower hypertension-related mortality: a prospective cohort study in French men.. Am J Clin Nutr.

[6] Andriambeloson E, Magnier C, Haan-Archipoff G, Lobstein A, Anton R (1998). Natural dietary polyphenolic compounds cause endothelium-dependent vasorelaxation in rat thoracic aorta.. J Nutr.

[7] Martin S, Andriambeloson E, Takeda K, Andriantsitohaina R (2002). Red wine polyphenols increase calcium in bovine aortic endothelial cells: a basis to elucidate signalling pathways leading to nitric oxide production.. Br J Pharmacol.

[8] Bernatova I, Pechanova O, Babal P, Kysela S, Stvrtina S (2002). Wine polyphenols improve cardiovascular remodeling and vascular function in NO-deficient hypertension.. Am J Physiol Heart Circ Physiol.

[9] Pechanova O, Rezzani R, Babal P, Bernatova I, Andriantsitohaina R (2006). Beneficial effects of Provinols: cardiovascular system and kidney.. Physiol Res.

[10] Ritz MF, Ratajczak P, Curin Y, Cam E, Mendelowitsch A (2008). Chronic treatment with red wine polyphenol compounds mediates neuroprotection in a rat model of ischemic cerebral stroke.. J Nutr.

[11] Pechanova O, Bernatova I, Babal P, Martinez MC, Kysela S (2004). Red wine polyphenols prevent cardiovascular alterations in L-NAME-induced hypertension.. J Hypertens.

[12] Sakamoto K (2008). Silencing metabolic disorders by novel SIRT1 activators.. Cell Metab.

[13] Baur JA, Pearson KJ, Price NL, Jamieson HA, Lerin C (2006). Resveratrol improves health and survival of mice on a high-calorie diet.. Nature.

[14] Walker HA, Dean TS, Sanders TA, Jackson G, Ritter JM (2001). The phytoestrogen genistein produces acute nitric oxide-dependent dilation of human forearm vasculature with similar potency to 17beta-estradiol.. Circulation.

[15] Räthel TR, Leikert JF, Vollmar AM, Dirsch VM (2005). The soy isoflavone genistein induces a late bus sustained activation of the endothelial nitric oxide-synthase system in vitro.. Br J Pharmacol.

[16] Vera R, Sanchez M, Galisteo M, Villar IC, Jimenez R (2007). Chronic administration of genistein improves endothelial dysfunction in spontaneously hypertensive rats: involvement of eNOS, caveolin and calmodulin expression and NADPH oxidase activity.. Clin Sci (Lond.).

[17] Li L, Hisamoto K, Kim KH, Haynes MP, Bauer PM (2007). Variant estrogen receptor-c-Src molecular interdependence and c-Src structural requirements for endothelial NO synthase activation.. Proc Natl Acad Sci U S A.

[18] Andriambeloson E, Kleschyov AL, Muller B, Beretz A, Stoclet JC (1997). Nitric oxide production and endothelium-dependent vasorelaxation induced by wine polyphenols in rat aorta.. Br J Pharmacol.

[19] Kobayashi M, Inoue K, Warabi E, Minami T, Kodama T (2005). A simple method of isolating mouse aortic endothelial cells.. J Atheroscler Thromb.

[20] Klinge CM, Wickramasinghe NS, Ivanova MM, Dougherty SM (2008). Resveratrol stimulates nitric oxide production by increasing estrogen receptor alpha-Src-caveolin-1 interaction and phosphorylation in human umbilical vein endothelial cells.. Faseb J.

[21] Miller VM, Mulvagh SL (2007). Sex steroids and endothelial function: translating basic science to clinical practice.. Trends Pharmacol Sci.

[22] Martin S, Giannone G, Andriantsitohaina R, Martίnez MC (2003). Delphinidin, an active compound of red wine, inhibits endothelial cell apoptosis via nitric oxide pathway and regulation of calcium homeostasis.. Br J Pharmacol.

[23] Klinge CM, Blankenship KA, Risinger KE, Bhatnagar S, Noisin EL (2005). Resveratrol and estradiol rapidly activate MAPK signaling through estrogen receptors alpha and beta in endothelial cells.. J Biol Chem.

[24] Agouni A, Lagrue-Lak-Hal AH, Mostefai HA, Tesse A, Mulder P (2009). Red wine polyphenols prevent metabolic and cardiovascular alterations associated with obesity in Zucker fatty rats (Fa/Fa).. PLoS ONE.

[25] Baron-Menguy C, Bocquet A, Guihot AL, Chappard D, Amiot MJ (2007). Effects of red wine polyphenols on postischemic neovascularization model in rats: low doses are proangiogenic, high doses anti-angiogenic.. Faseb J.

[26] Agouni A, Mostefai HA, Porro C, Carusio N, Favre J (2007). Sonic hedgehog carried by microparticles corrects endothelial injury through nitric oxide release.. Faseb J.

[27] Mostefai HA, Meziani F, Mastronardi ML, Agouni A, Heymes C (2008). Circulating microparticles from patients with septic shock exert protective role in vascular function.. Am J Respir Crit Care Med.

[28] Verdonk ML, Chessari G, Cole JC, Hartshorn MJ, Murray CW (2005). Modeling water molecules in protein-ligand docking using GOLD.. J Med Chem.

