# Supplementary material for: Estrogen Receptor Alpha as a Key Target of Red Wine Polyphenols Action on the Endothelium
Source: PLoS One. 2010 Jan 1;5(1):e8554. doi: 10.1371/journal.pone.0008554 (PMC2796721; doi:10.1371/journal.pone.0008554)
Supplement: Methods S1 — (0.04 MB DOC) [file pone.0008554.s001.doc]

Methods

**Products**

Provinols^TM^ was obtained from Société Française des Distilleries (Vallon Pont d'Arc, France). The composition is, in mg/g of dry powder: 480 proanthocyanidins, 61 total anthocyanins, 19 free anthocyanins, 38 catechin, 18 hydroxycinnamic acids, 14 flavonols and 370 polymeric tannins. Delphinidin was purchased from Extrasynthèse (Genay, France) with purity > 97%, and PPT and E_2_ were from Sigma-Aldrich (St Quentin Fallavier, France).

For cellular experiments Provinols^TM^, delphinidin, PPT and E_2_ were dissolved in pure dimethylsulfoxide and then diluted in the cell culture medium to a final dimethylsulfoxide concentration of 1%.

For animal feeding, Provinols^TM^ was incorporated directly in animal food. The concentration of polyphenols present in the plasma with the dose of Provinols^TM^ (20 mg/kg) used in the in vivo was comparable to the concentration at which RWPCs produced endothelium-dependent relaxation in rat aortic rings because only 5% to 10% of polyphenols could be absorbed in the digestive tract, as discussed in our previous manuscript [1].

**Vascular reactivity**

The university of Angers ethical committee approved the present protocol. All animal studies were carried out using approved institutional protocols and were conformed the Guide for the Care and Use of Laboratory Animals published by US National Institutes of Health (NIH Publication No. 85-23, revised 1996). Female ERα Wild Type or Knock-Out mice were ovariectomized at the age of 12 weeks. After 7 days, mice were killed by cervical dislocation (first set of experiments (Figure 1)), Aortic rings were isolated and mounted on a wire myograph filled with Krebs solution with the following composition in mM: NaCl 130, NaHCO_3_ 14.9, KCl 3,7, KH_2_PO_4_ 1.2, MgSO_4_.7H_2_O 1.2, CaCl_2_.H_2_O 1.6, glucose 11 (37°C, 95% O_2_-5% CO_2_). Mechanical activity was recorded isometrically by a force transducer (Danish Myo Technology, Aarhus, Denmark). Concentration-response curves were constructed by cumulative application of PPT (0.01-10 μM), E_2_ (0.01-10 μM), Provinols^TM^ (0.1-100 g/L) or delphinidin (0.1-10 g/L), to vessels with functional endothelium pre-contracted with U46619 at 80% of their maximal response.

For a second set of experiments (Figure 5), ovariectomized mice were separated in four groups: WT and KO mice fed either with control diet or diet containing Provinols^TM^, for 2 weeks. After treatment, mice were killed by cervical dislocation. Aortic rings were isolated and mounted on a wire myograph filled with Krebs solution as described above. Concentration-response curves were constructed by cumulative application of serotonin (5-HT) (0.01-10 µM) or acetylcholine (Ach) (0.1 nM-10 µM) to vessels with functional endothelium pre-contracted with U46619 at 80% of their maximal response.

**Cell culture**

EaHy 926 was a generous gift from Dr Edgell (Pathology and Laboratory Medicine Department, University of North Carolina, USA) [2]. EaHy 926 endothelial cell line was maintained in culture in medium culture [DMEM, Ham’s F-12 1:1 (Lonza, Walkersville, MD), 1 % L-Glutamine, 1 % HAT (Sigma-Aldrich), 1 % NEAA, 1 % Na-Pyruvate and 1 % Streptomycine/Penicilline] supplemented with 10 % of FBS (Lonza).

Primary endothelial cells (EC) were isolated from aortas taken ERα WT and KO mice. The extraction method was adapted from Kobayashi’s protocol [3]. ECs were cultured in growth medium EBM-2 (Lonza) supplemented with 5% FBS. ECs were used until their 3^rd^ – 4^th^ passage.

Cells were stimulated for 10 minutes at maximal effective concentration with each treatment (Provinols^TM^, delphinidin, PPT or E_2_), for NO detection (EaHy and ECs) or lysed for protein expression studies by western blot analysis (EaHy).

**RNA interference and transient transfection**

In order to silencing ERα, siRNA duplexes specific for human ERα and control, non-silencing, siRNA were obtained from Santa Cruz Biotechnology (Santa Cruz, CA). Transient transfection of EaHy 926 endothelial cells was performed according to the manufacturer's protocol and as previously described [4]. Briefly, cells were seeded in 6-well plates, grown for 24 hours (60% confluence), and then transiently transfected with 100 nM of ERα-specific or control siRNA using the transfection reagent provided, which also served as control without siRNA. Medium was replaced 24 hours later by fresh medium and cells were grown for additional 24 hours, prior to either western blot analysis of ERα expression or functional studies. Cells were treated with Provinols^TM^, delphinidin, PPT or E_2_ for NO measurements or lysed for protein expression studies by western blot analysis.

**NO and O_2_^-^ spin trapping and electronic paramagnetic resonance (EPR) studies**

Detection of NO production was performed using the technique with Fe^2+^ diethyldithiocarbamate (DETC, Sigma-Aldrich) as spin trap. Briefly, after treatment, cells or mesenteric arteries were stimulated for NO production by their incubation for 30 min in Krebs–Hepes buffer containing: BSA (20.5 g/L), CaCl_2_ (3 mM) and L-Arginine (0.8 mM). NaDETC (3.6 mg) and FeSO_4_-7H_2_O (2.25 mg, Sigma-Aldrich) were separately dissolved under nitrogen gas bubbling in 10 ml volumes of ice-cold Krebs–Hepes buffer. The solutions were rapidly mixed to obtain a pale yellow-brown opalescent colloid Fe(DETC)_2_ solution (0.4 mM), which was used immediately to incubate cells for 45 minutes at 37 °C. After incubation spin trap was removed, cells were scrapped in physiological salt solution, mesenteric arteries were immersed in physiological salt solution and both frozen in liquid nitrogen.

NO measurement was performed on a table-top x-band spectrometer Miniscope (Magnettech, MS200, Berlin, Germany). Recordings were made at 77 °K, using a Dewar flask. Instrument settings were 10mW of microwave power, 1mT of amplitude modulation, 100 kHz of modulation frequency, 150 s of sweep time and 5 scans. Signals were quantified by measuring the total amplitude, after correction of baseline as done previously [5].

For O_2_^−^ spin-trapping, mesenteric arteries were dissected and allowed to equilibrate in deferoxamine-chelated Krebs-Hepes solution containing 1-hydroxy-3methoxycarbonyl-2,2,5,5-tetramethylpyrrolidin (CMH, Noxygen, Denzlingen, Germany) (500 µM), deferoxamin (25 µM, Sigma-Aldrich) and DETC (5 µM, Sigma-Aldrich) under constant temperature (37°C) for 60 minutes. Then, they were frozen in liquid N_2_ and analyzed in on a table-top x-band spectrometer Miniscope. Recordings were made at 77 °K, using a Dewar flask. Instrument settings were 10mW of microwave power, 1mT of amplitude modulation, 100 kHz of modulation frequency, 60 s of sweep time and 5 scans. Signals were quantified by calculating mean of the total amplitude of the three peaks, after correction of baseline as done previously [5].

**Western Blotting**

After treatments, cells were homogenized and lysed. Proteins (40 µg) were separated on 10 % SDS-PAGE electrophoresis gel. Blots were probed with ERα (Santa Cruz), phospho-caveolin-1, phospho-eNOS Ser 1177, phospho-ERK p42/44 and phospho-Src antibodies (1:500, Cell Signalling, Danvers, MA). A polyclonal anti-mouse ß-actin antibody (1:2000, Sigma-Aldrich) was used to visualize protein gel loading. Bound antibodies were detected with a secondary peroxidase-conjugated anti-rabbit or anti-mouse IgG (Promega, Charbonnieres, France). The blots were visualized using the enhanced chemiluminescence system (ECL Plus, Amersham Biosciences, Piscataway, NJ) or for phosphorylated proteins using the Super Signal West Femto (Thermo Scientific Pierce, Brebières, France) and quantified by densitometry and normalized to β-actin expression.

**Binding test**

Binding assay was performed by CEREP (Paris, France). Briefly, purified receptor (13 nM) is mixed with 1 nM fluorescein-labeled estrogen (Fluormone^TM^ ES2) and the complex incubated for 2 hours at room temperature in the absence or presence of delphinidin in a buffer containing 100 mM K_2_HPO_4_/KH_2_PO_4_ (pH 7.4), 10 µg/ml bovine gamma globulin and 0.02% NaN_3_. Nonspecific binding is determined in the presence of E_2_ (10 µM). Following incubation, the fluorescence polarization is measured at λex = 485 nm and λem = 535 nM using a microplate reader (Ultra, Tecan). The results are expressed as a percent inhibition of the control Fluormone specific binding. Compound interference with the detection method due to autofluorescence is checked by measurements at the same wavelengths in the incubation buffer before the addition of the Fluormone/receptor complex. The standard reference compound is E_2_, which is tested in each experiment at several concentrations to obtain a competition curve from which its IC_50_ is calculated.

**Docking**

3-D coordinates of delphinidin were generated in mol2 file format (TRIPOS Associates, St.Louis, USA) from 2-D sketches using Corina (Molecular Networks GmBH, Erlangen, Germany). Atomic coordinates of the ERα (1 a52 entry) in complex with E_2_ [6] were extracted from the Protein Data Bank [7]. Hydrogen atoms were then manually added to optimize first intermolecular hydrogen bonds with the ligand, and then the intramolecular hydrogen bond network. The ligand and water molecules were then removed and ligand-free protein coordinates saved in mol2 file format. Delphinidin was docked using default settings of the GOLD4.0 program [8]. The binding site cavity was defined from any amino acid present in a 6.5-Å radius-sphere centered on E_2_ in the original pdb coordinates. A maximum of 10 docking poses were generated and rescored with our in-house topological scoring function [9] registering the similarity of protein-ligand interaction with that occurring in the 1a52 template. The docking solution with the highest similarity score was last saved for visual inspection.

**Data analysis**

Data are represented as mean ± SEM, n represents the number of experiences. Statistical analyses were performed by a one way analysis of variance (ANOVA), and Mann-Whitney U tests or ANOVA for repeated measures and subsequent Bonferroni post hoc test. *P* < 0.05 was considered to be statistically significant.

**References**

1. Diebolt M, Buchner B and Andriantsitohaina R (2001) Wine plyphenols decrease blood pressure, improve NO vasodilatation, and induce gene expression. Hypertension 38: 159-165.

2. Suggs JE, Madden MC, Friedman M, Edgell CJ (1986) Prostacyclin expression by a continuous human cell line derived from vascular endothelium. Blood 68: 825-829.

3. Kobayashi M, Inoue K, Warabi E, Minami T and Kodama T (2005) A simple method of isolating mouse aortic endothelial cells. J Atheroscler Thromb 12: 138-142.

4. Agouni A, Mostefai HA, Porro C, Carusio N, Favre J, et al. (2007) Sonic hedgehog carried by microparticles corrects endothelial injury through nitric oxide release. Faseb J 21: 2735-2741.

5. Agouni A, Lagrue-Lak-Hal AH, Mostefai HA, Tesse A, Mulder P, et al. (2009) Red wine polyphenols prevent metabolic and cardiovascular alterations associated with obesity in Zucker fatty rats (Fa/Fa). PLoS ONE 4: e5557.

6. Tanenbaum DM, Wang Y, Williams SP, Sigler PB (1998) Crystallographic comparison of the estrogen and progesterone receptor's ligand binding domains. Proc Natl Acad Sci U S A 95: 5998-6003.

7. Berman HM, Westbrook J, Feng Z, Gilliland G, Bhat TN, et al. (2000) The Protein Data Bank. Nucleic Acids Res 28: 235-242.

8. Verdonk ML, Chessari G, Cole JC, Hartshorn MJ, Murray CW, et al. (2005) Modeling water molecules in protein-ligand docking using GOLD. J Med Chem 48: 6504-6515.

9. Marcou G, Rognan D (2007) Optimizing fragment and scaffold docking by use of molecular interaction fingerprints. J Chem Inf Model 47: 195-207.
